# Neutrophil Extracellular Traps (NETs) and Vasculitis

**DOI:** 10.7150/ijms.53728

**Published:** 2021-02-03

**Authors:** Borros Arneth, Rebekka Arneth

**Affiliations:** 1 Institute of Laboratory Medicine and Pathobiochemistry, Molecular Diagnostics, University Hospital of the Universities of Giessen and Marburg UKGM, Justus Liebig University Giessen, Giessen, Germany.; 2 Clinic of Nephrology, Internal Medicine, University Hospital of the Universities of Giessen and Marburg UKGM, Justus Liebig University Giessen, Giessen, Germany.

**Keywords:** neutrophil extracellular traps, vasculitis

## Abstract

**Background:** Neutrophil extracellular traps (NETs) have been implicated in host immune responses. Attempts have been made to examine how NETs affect the pathogenesis of complications such as autoimmune and vascular disorders.

**Aim:** This study aimed to explore the relationship between NETs and vasculitis.

**Material and Methods:** The current study entailed the searching of PsycINFO, PubMed, Web of Science, and CINAHL for articles related to the research topic. The search terms and phrases included “vasculitis,” “NETs,” “neutrophil extracellular traps,” “NETosis,” and “pathogenesis.” The search was limited to articles published between 2009 and 2019.

**Results:** Researchers have shown that NETs contribute to the pathogenesis of vasculitis through different mechanisms and processes, including renal failure and vascular damage. The protective effects of NETs have also been highlighted.

**Discussion:** Overall, some scholars have shown the effectiveness of using DNase I and the PAD4 inhibitor Cl-amidine to treat vasculitis by restricting NET formation. However, observations have been noted in only animal experimental models.

**Conclusion:** Neutrophil hyperactivity and its role in vasculitis are not yet fully understood. More studies aiming to determine the accurate function of NETs in vasculitis pathogenesis, particularly in humans, should be undertaken. Intensive research on NETs and vasculitis can increase the knowledge of medical practitioners and contribute to the development of new treatment methods to enhance patient outcomes in the future.

## Introduction

Neutrophils comprise approximately 57% of circulating white blood cells and are regarded as the most important effector cells in the innate immune system 1. Neutrophils are normally recognized by their lobulated nuclei and short lifespans compared to those of other subpopulations of white blood cells 2,3. When a pathogen enters the body, neutrophils act as the first line of response. They are directed to the infection or inflammation site, where they recruit and activate other types of immune cells to initiate the appropriate response 4-6. While exerting their primary defense response, neutrophils can phagocytose disease-causing pathogens and release granules that contain proteases and antimicrobial peptides 7,8. Researchers have recently reported that neutrophils can attack and restrain pathogens directly through the release of neutrophil extracellular traps (NETs) in a process commonly referred to as NETosis 3. According to Linkermann et al., NETosis involves the release of extracellular DNA by host neutrophils 3. Initially, NETosis was considered to coincide with the death of cells in the body, but recent investigations have revealed that it is a regulated cell death pathway 9. Interestingly, NETosis is a caspase-independent process 10. Researchers have yet to reach a consensus on whether NETosis is an anti-inflammatory or immunogenic process because it involves diverse pathways, some of which are still being studied 10. The situation is further complicated by the fact that NET formation can take place even in the absence of necrosis. In other words, the development of NETs in humans occurs independently without the signaling of RIPK3 and MLKL, and the process is therefore independent of necroptosis 11,12. Nevertheless, NETosis is considered a critical process that helps eliminate disease-causing pathogens.

Several previous studies have focused on NET formation as a process by which the body identifies and kills invading pathogens. In such studies, researchers noted that NETosis appeared to be a form of programmed cell death that was primarily initiated by nicotinamide adenine dinucleotide phosphate (NADPH) oxidase activation 13,14. NADPH oxidase activation could be linked to nuclear membrane breakdown, chromatin decondensation, or the mixing of granule constituents and chromatin. Furthermore, NETosis is reported to be largely dependent on other molecules and substances, such as neutrophil elastases, peptidyl arginine deiminase (PAD), and myeloperoxidase (MPO) 15. More recently, there have been sustained attempts to examine the link between NETosis and specific conditions, such as cancer, autoimmune disorders, and arteriosclerosis 16. This interest stems from the realization that neutrophils are not the only subpopulation of cells capable of releasing extracellular traps; research evidence shows that other cells, such as mast cells, monocytes, and basophils, also have this ability even if it is rarely used 17. Furthermore, the realization that NET formation can occur independently of cell death has motivated researchers to conduct further research.

The possible association between NET formation and vasculitis has also been explored in recent studies. Systemic vasculitis refers to a broad spectrum of disorders characterized by idiopathic vascular inflammation 18 and is categorized based on the size of the affected vessels. Irrespective of the nature of the disorder, both its pathogenesis and prognosis are potentially associated with NET formation 18. Therefore, further investigations are required to understand the underlying correlation between NETs and vasculitis and possible therapeutic strategies for treating this disease. This systematic review aimed to explore the association between NETs and vasculitis.

## Methodology

**Figure 1:**
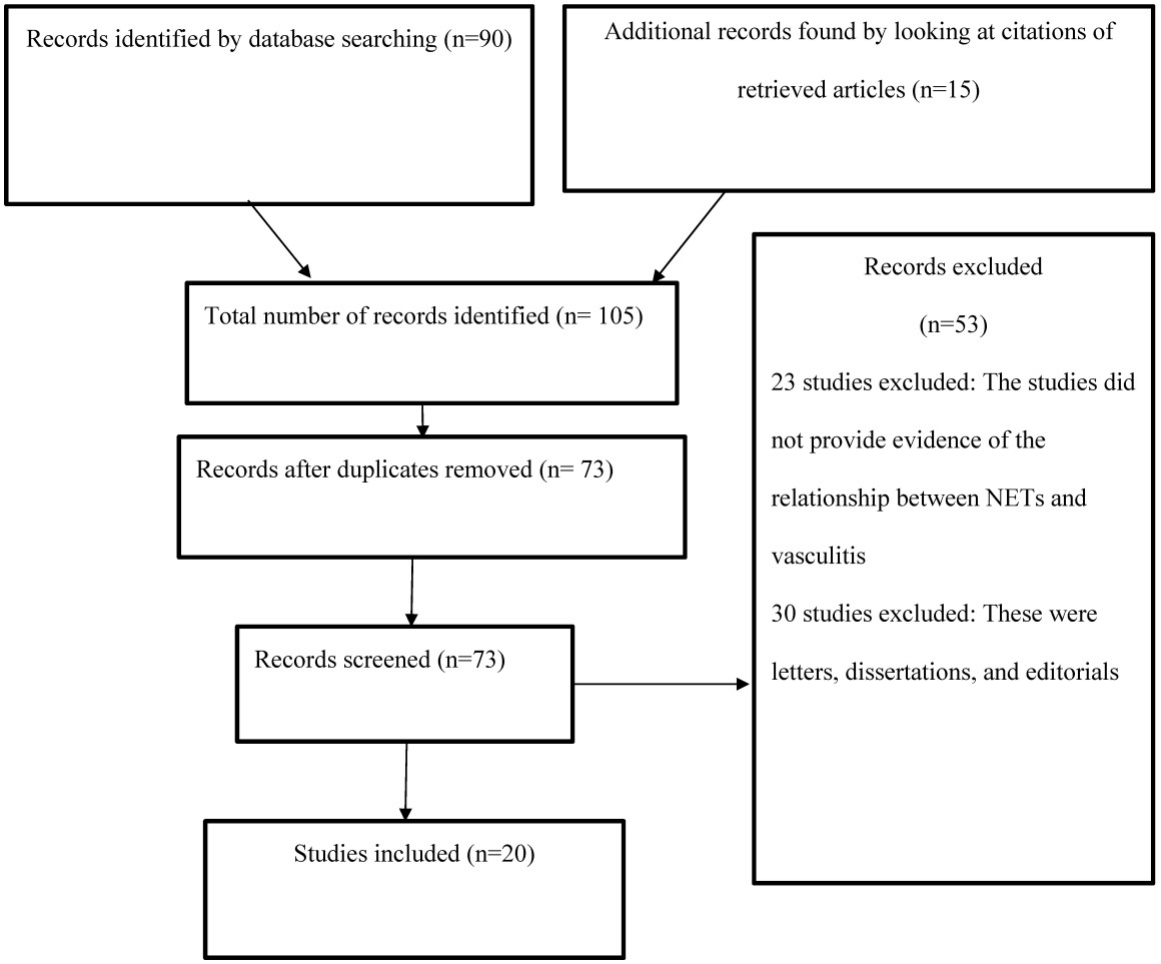
PRISMA flow diagram.

The current study examined the relationship between NETs and vasculitis by reviewing the existing body of research evidence, which entailed analyzing the results of investigations related to the topic of interest. The articles used were obtained from PsycINFO, PubMed, Web of Science, and CINAHL using the search terms and phrases “vasculitis,” “NETs,” “neutrophil extracellular traps,” “NETosis,” and “pathogenesis.” The search was limited to articles published in the four electronic databases between 2009 and 2019. The abstracts of the available articles were carefully reviewed to determine their quality and appropriateness (Figure 1).

## Results

At the end of the search and review process, the final list of articles consisted of animal model studies, clinical trials, experimental studies, and clinical reviews (Table 1). The articles were carefully examined to explore the relationship between NETs and vasculitis. An analysis of studies revealed that NETs are considered an important component of the innate immune system that helps fight disease 1,14,16. The evidence gathered from the studies highlights the significant role of NETs in the adaptive immune response and their possible interaction with lymphocytes and antigen-presenting cells in lymph nodes and sites of inflammation 16,18. Other studies revealed that NETs can induce antibody production and affect the actions of other molecules, such as proproliferative ligands 19,20. Therefore, the analysis and elucidation of NETosis and regulatory mechanisms that affect innate immunity in patients with vasculitis can provide novel therapies for disease management.

### Vasculitis and increased NETosis

The study of vasculitis pathogenesis has provided opportunities for the development of new treatment strategies. Vasculitis is a term used to indicate a broad spectrum of diseases that affect blood vessels 40, including anti-neutrophil cytoplasmic antibody (ANCA)-associated vasculitis (AAV, small-vessel vasculitis), Takayasu arteritis (large-vessel vasculitis), and giant cell arteritis 41. These disorders can be triggered by various environmental factors and infectious pathogens that lead to vascular injury. A review of previous studies shows that vasculitis usually develops when host antibodies mistake blood vessel proteins for foreign bodies or pathogens 42. In such instances, immune cells attack and destroy blood vessel cells. In other studies, researchers have noted that extravascular granulomatosis is a primary trigger of vasculitis in humans 43. Therefore, the pathogenesis of vasculitis may vary depending on the type and on the triggers involved 21. Furthermore, the disease manifestations are diverse and may include renal failure, aneurysms, visual disturbances, and glomerulonephritis. In some patients, vasculitis can lead to life-threatening complications such as pulmonary arterial hypertension, coronary heart disease, and thrombosis 44. Consequently, there is a growing desire to explore the causes of vasculitis and to identify factors, processes, and biomarkers that can be targeted in the development of safer and more effective therapies.

The roles of NETs in the development and progression of vasculitis have been explored in previous studies. Research evidence showed that dying neutrophils around the walls of small vessels are a hallmark of AAV pathogenesis 45. Additionally, they can attract other immune cells to the sites of inflammation or injury through the production of chemokines and cytokines 45. However, it is not always easy to differentiate between the effects of degranulation induced by NETs and neutrophil-related cell death, as researchers contend that increased NETosis characterizes active ANCA-associated vasculitis. Furthermore, recent investigations have revealed that blood and affected tissue samples from patients with ANCA-associated vasculitis, Takayasu arteritis, and giant cell arteritis contain higher levels of NET remnants than those obtained from healthy individuals 45. Studies have shown that PR3-ANCAs or MPO-ANCAs are found in most patients with vasculitis, and these forms of ANCAs are the primary determinants of AAV pathogenesis 46. Nonetheless, other researchers have stated that healthcare practitioners and clinical experts need to be aware that NETs can also be mitochondrially derived and thus lack histones 47.

The first clear evidence of increased NET formation in patients with vasculitis was based on kidney biopsy samples 22,48. Later, researchers showed that NETs also exist in skin specimens and thrombus samples obtained from people diagnosed with conditions such as Henoch-Schonlein purpura, granulomatosis with polyangiitis, microscopic polyangiitis, and eosinophilic granulomatosis 49-51. In addition, patients with ANCA-negative MPA were reported to have increased NET levels in nerve biopsies than counterparts who did not have the disease 23,52. The differences in NET and NET fragment levels indicate the possible role of NETosis in the pathogenesis and progression of vasculitis. *In vitro* studies have shown that patients with ANCA-associated vasculitis are more likely to experience spontaneous NETosis than healthy controls 53,54. Furthermore, such patients are more likely to be affected by NET-inducing stimuli than those not suffering from this disease. In autoimmune studies, researchers have reported that NETosis can be negatively regulated by the interaction between neutrophil semaphorin D on neutrophils and plexin B2 on endothelial cells. Moreover, recombinant plexin B2 can inhibit NETosis in human cells 54-56. These results provide a basis for understanding the possible links between NETs and the pathogenesis of ANCA-associated vasculitis.

Recent *in vitro* studies have shown that autoantibodies and serum immune complexes obtained from patients with ANCA-associated vasculitis are capable of inducing NETosis 24-26,56-59. In addition, IgG from these patients can stimulate NETosis 24-26,56-59. It has been reported that antibodies against proteinase 3 (PR3) are present in the sera of patients with Wegener's granulomatosis 24,57. Furthermore, antibodies against MPO are present in the sera of patients with polyarteritis nodosa 24-26,56-59. However, the underlying mechanisms that link NETosis to the development of these diseases remain unclear. A recent study by Ma et al. reported that NET formation in patients with conditions such as vasculitis could be affected by MPO-ANCA IgG and PR3-ANCA IgG 58. Recent investigations showed that the mechanisms by which MPO-ANCA IgG causes NETosis in people with vasculitis could be linked to the degree of antibody affinity rather than to the antibody levels 25,26,57. Furthermore, renal biopsy investigations have revealed that people with high-affinity MPO-ANCA IgG tend to show increased levels of NETosis 59. Interestingly, some studies have documented that IgG-depleted sera from patients without ANCAs can also induce NETosis 27,59,60. The results of such studies question the role of IgG in influencing NETosis in patients with vasculitis.

The link between NET formation and ANCA-associated vasculitis has also been examined by analyzing how immune complexes with IgG can induce NETosis by activating neutrophils through the crosslinking of the Fcγ receptor IIIb 131 in patients with MPO-ANCA-associated microscopic polyangiitis 27,28. Recent studies have shown that aggregated immune complexes are receptor-specific and enhance the NETosis rate in patients diagnosed with conditions such as vasculitis and systemic lupus erythematosus (SLE) 28,61. Other studies have noted that IgA immune complexes from synovial and plasma fluid samples can induce NETosis through Fc alpha receptor I 29. The results indicate the pathogenesis of small-vessel leukocytoclastic vasculitis since IgA immune complex deposition is an indication of this disease. In addition, the findings are supported by recent investigations revealing that samples from patients with SLE and rheumatoid arthritis had high levels of PR3-ANCA IgA depending on the disease severity 30.

In addition to effects on neutrophil activation, MPO-ANCAs and PR3-ANCAs can induce NETosis in patients suffering from SLE and AAV 57. The exact mechanism underlying the activation process remains unclear, but researchers believe that it requires the binding of autoantibodies to Fc receptors and PR3/MPO on the neutrophil surface. Recent research shows that neutrophil activation by MPO-ANCAs during NETosis can increase ROS production 57. A review of evidence from *in vitro* studies showed that neutrophils from patients with vasculitis are usually more readily activated by ANCAs than those from healthy people 26,58. Moreover, previous research revealed that vasculitis patients are more likely to show increased PR3 expression; this phenomenon can be explained by disruptions in the epigenetic silencing of the MPO and PR3 genes in affected patients. The epitope affinity and specificity of the involved autoantibodies are important concerning the development of AAV 60; the primary hypothesis is that neutrophil activation by ANCAs and the subsequent induction of NETs are affected by the affinity of the autoantibodies.

The current review makes it evident that NETs play a key role in the initiation and progression of AAV. There is compelling research evidence that NETs are directly involved in vessel inflammation 27,28; this process entails endothelial cell damage and complement system activation. In some instances, NETs are indirectly involved in the pathogenesis of AAV through the generation of MPO-ANCAs and PR3-ANCAs 61. The vicious cycle affects the development of AAV by activating neutrophils in the body. Importantly, ANCA-linked pathogenicity is influenced by the epitope specificity and affinity of autoantibodies 29. Furthermore, NET formation in patients with AAV may be increased by drugs, age-associated epigenetic changes, and infections. The increased NET formation should be balanced by clearance mechanisms, such as those involving DNases and autoantibodies with ANCA specificity 29,30. The data imply that under unfavorable conditions, people develop pathogenic autoantibodies that can activate neutrophils, creating a vicious cycle that causes vessel wall inflammation and AAV.

Another interesting reported finding regarding the link between NETs and vasculitis is that neutrophils obtained from people suffering from AAV are less likely to undergo apoptosis than those obtained from healthy individuals; this result suggests that such neutrophils are more susceptible to other forms of cell death depending on their cellular state and contextual factors 45. For instance, it has been reported that neutrophils from patients with AAV release NETs more spontaneously than those from healthy people 22,62,63. In addition, low-density granulocytes (LDGs), a subpopulation of neutrophils, show a stronger association than normal-density neutrophils with the increased spontaneous production of NETs 64. LDGs are categorized as a primary source of NETs in patients diagnosed with AAV 63. Furthermore, recent studies on LDGs show that they can promote the increased expression of mRNAs associated with different alarmins and immunostimulatory bactericidal proteins more than normal-density neutrophils 45,65. Such findings provide a basis for examining and understanding the pathogenesis of AAV and the possible link between this disease and NETs.

NETs can directly or indirectly cause vascular damage and worsen the symptoms of ANCA-associated vasculitis. For instance, the release of noxious molecules such as degrading enzymes can induce endothelial cell apoptosis and cause deterioration of the basement membrane 30,66. Histones may be released during NETosis and have toxic effects on endothelial cells 66. Although available research suggests that NETs can be phagocytized by endothelial cells, excessive amounts of NETs may promote vascular leakage and hinder interactions among endothelial cells 31. Moreover, NET formation is associated with the transformation of endothelial cells to mesenchymal cells, which interferes with normal glomerular function 30. When the resulting vasculitis is not managed, the alternative complement pathway can be activated, resulting in tissue fibrosis 31,32,67,68.

Finally, the presence of renal injury among AAV patients is also regarded as an indicator of the role of NETs in disease development. Research shows that patients suffering from small-vessel vasculitis are likely to experience tubulointerstitial nephritis and glomerulonephritis 33,68. Recent investigations have indicated that NETs are usually present in glomeruli 68. Furthermore, researchers have used animal model studies to show that NET formation can cause glomerular vasculitis by activating anti-glomerular basement membrane (GBM) antibodies 32,33,36,68. In addition, NET formation is believed to lead to tubulointerstitial injury and epithelial tubular cell hypoxia. In some instances, NETs release histones from endothelial cells, thus causing necrosis and necroinflammation 69-72. Moreover, NETs can worsen tubulointerstitial injury and compromise glomerular blood flow. The findings show that NETs contribute to the development of vasculitis by causing renal injury and increasing the risk of hemorrhage (Table 2).

### Protective effect of NETs

Recent research projects have shown that NETs can protect patients from the adverse effects of vasculitis 18. One experimental study reported that saliva could induce the formation of NETs. However, the induction capacity was diminished in patients suffering from a form of vasculitis known as Bechet's disease, which is usually characterized by acute to chronic genital and mouth ulcers 18. In this study, the authors argued that the absence of NETs diminished protection against bacteria in the mucous membrane, thus promoting ulcer formation. In another animal model study, NETs were shown to provide a platform for degrading proinflammatory mediators 71,72. Under some circumstances, NETs tend to impair GM-CSF/IL-4-linked dendritic cell differentiation and promote the activation of macrophage phenotypes 73. Macrophage cell populations help reduce inflammation and promote autoantibody production in response to the disease-causing pathogens in question 56. Significant amounts of research evidence show that NETs may contain alarmins such as HMGB1 and LL39 and enhance the rate of autoantibody production in people with vasculitis 34,73,74. The actual mechanisms by which these processes protect against vasculitis need to be studied further to determine their therapeutic potential.

## Discussion

Vasculitis is a severe condition that can lead to adverse outcomes, including death, if not well managed 34,75. Over the years, attempts have been made to understand and study the possible causes of vasculitis and the underlying pathogenesis 75,76. Investigations have shown that this disease is characterized by the abnormal attack of blood vessel cells following foreign pathogen invasion 35,36. Evidence gathered in this study reveals that NETs can lead to the emergence and progression of vasculitis through a wide range of pathways, such as vascular damage and renal complications 34. More recently, however, NETs were reported to potentially have protective effects, reducing the risk of vasculitis and lessening the severity of symptoms 34. Therefore, NETs can be regarded as an important biomarker for detecting and monitoring vasculitis activity. Furthermore, they provide an avenue for developing new therapeutic strategies.

For instance, several approaches for treating vasculitis that involve blocking the adverse effects of NETs have been examined *in vivo*. Antihistamine therapy is one technique that works by promoting the production of antihistamine antibodies 34,36. Heparin and activated protein C-based treatment have been shown to be effective in the management of anti-GBM-induced vasculitis 31. Similar results were reported in studies that analyzed the possibility of using the PAD4 inhibitor Cl-amidine to treat vasculitis, as this inhibitor blocks histone citrullination during NETosis 37,77. Additionally, mouse model studies involving the use of PAD4 to manage vasculitis have shown that the technique could effectively minimize the generation of NET-mediated inflammation 34. This process is hypothesized to entail controlling ROS signaling and NETosis using tetrahydroisoquinoline, a compound that can regulate PAD4 and NETosis, via inhibition of the latter and interfering with ROS production 34.

Other researchers have noted that DNA degradation, ROS scavenging, and mitochondrial ROS inhibition might also help manage vasculitis 34,77. The inhibitory processes are not well understood but are believed to be related to the upregulation of superoxide dismutase 3, the inhibition of mitochondrial ROS, and neutrophil phagocytosis 38,39,70,78. Based on studies on the protective effects of NETs, researchers have hypothesized that compounds such as the anti-thyroid drug propylthiouracil (PTU) could help in the management of vasculitis by promoting autoantibody production to neutralize disease-causing pathogens and the treatment of hyperthyroidism 34,79. PTU can induce the formation of NETs that hinder DNase I degradation, thus reducing disease severity 34,39,75,80.

Nonetheless, despite all these outcomes, minimal attention has been directed at exploring the role of NETs as biomarkers of vasculitis activity. In addition, only a few studies have investigated how NETs can be used to monitor disease progression and the treatment response 34. The present systematic review highlighted that the NET and NET-related protein levels were increased in samples from patients with vasculitis compared to those from healthy people. However, researchers and healthcare practitioners have yet to develop an assay based on NET biomarkers that can be used to monitor vasculitis progression in the clinic 34. The situation is further complicated by the existence of a standard approach for measuring NET levels in patients 34,35,76. Nevertheless, there is a need to carry out further research on the utility of NETs as biomarkers for vasculitis and to determine the measures and parameters necessary to analyze treatment responses.

## Conclusion

From the review, it is evident that NETs play an essential role in the pathogenesis of vasculitis. The literature has shown that patients with vasculitis have more NET components than healthy people. Such an observation concludes the cause of chronic inflammation, which is one of the fundamental elements sought by medical practitioners when diagnosing vasculitis. It is currently believed that preventing the generation of structures can contribute to favorable clinical results for patients with vasculitis. Reports from studies have shown that currently, DNase I and the PAD4 inhibitor CI-amidine could impact the formation of NETs through restriction, which can help minimize the development of vasculitis. However, these observations have thus far been made using animal experimental models. As a result, in humans, neutrophil hyperactivity and its role in vasculitis are not yet fully understood. Therefore, more studies aiming to determine the accurate role of NETs in vasculitis pathogenesis should be undertaken, particularly in humans. Intensive research on NETs and vasculitis can increase the knowledge of medical practitioners and contribute to the development of new treatment methods to enhance patient outcomes in the future.

## Figures and Tables

**Figure 2:**
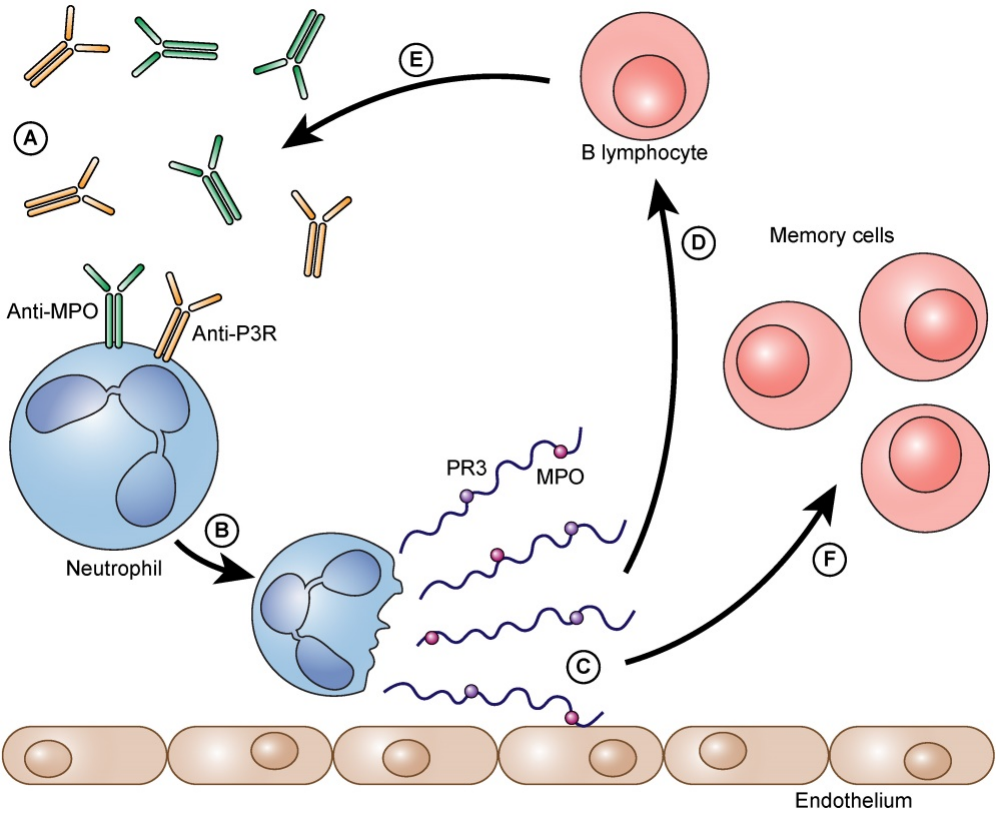
The process as a vicious circle. Auto-antibodies bind to neutrophil granulocytes and trigger NET formation (A). Neutrophils form NETs on the vascular endothelium (B). Immunogenic proteins such as PR3 and/or MPO are released (C). B- lymphocytes arrive and are immunized against these proteins (D). They form even more auto-antibodies against these proteins (PR3 and/or MPO) (E). The auto-antibodies that are formed can then, in turn, bind to neutrophils and can accelerate the further formation of NETs. Besides the immunized and thereby activated B lymphocytes also memory cells (memory B lymphocytes and memory T lymphocytes) are formed (F).

**Table 1:** Summary of the selected studies

Study	Design	Results
Lee et al. (2017) 10	Review	Interleukin-8, neutrophils, and anti-neutrophil cytoplasmic antibodies (ANCAs) are inflammatory molecules that play an important role in NET formation. The researchers noted that increased exposure to NTEs-related cascades and molecules could lead to systemic organ damage and to the development of autoimmune disorders. Further research is needed to explore how the NET formation process can be targeted to help treat autoimmune diseases.
Cornec et al. (2016) 21	Systematic review	The authors stated that ANCA-associated vasculitis is a disorder that is characterized by the inflammation of small and mid-sized blood vessels. Available research evidence shows that PR3-ANCAs and MPO-ANCAs can be diagnosed effectively by examining the specific genetic and epidemiologic factors associated with each of the conditions.
Tang et al. (2015) 22	Experimental study	Increasing research evidence suggests that NET formation is implicated in the development of ANCA-associated vasculitis. In addition, ANCA autoantibodies and anti-lysosomal membrane protein-2 (LAMP-2) antibodies have a critical pathogenic role in AAV. Targeting these molecules can help in the treatment and effective management of vasculitis.
Carmona-Rivera et al. (2017) 23	Review	NET formation is implicated in the development of idiopathic autoimmune disorders. The researchers noted that NETs that were generated by levamisole appeared to be toxic to the endothelial walls. In addition, they impair endothelium vasorelaxation. Finally, the study revealed that successful stimulation of neutrophil muscarinic receptors through the use of cholinergic agonists could lead to vascular damage.
Nishide et al. (2017) 24	Animal model studies	SEMA4D on the surface of neutrophils negatively control the neutrophil activation process. In patients with AAV, the proteolytic cleavage of the SEMA4D molecules can increase the neutrophil-related inflammatory responses in the body. Therefore, SEMA4D is a potential biomarker of AAV and a novel therapeutic target in the management of vasculitis.
Sha et al. (2016) 25	Review	Dysregulation of the NET formation process may lead to the development of ANCA-associated vasculitis. Research evidence shows that NET formation is usually characterized by autophagy. A review of previous studies showed that autophagy can be induced by ANCAs and can increase the rate of ANCA-associated NETosis.
Shida et al. (2016) 26	Clinical study	The combined stimulation of 1 µg/mL aLf. IgG and 10 nM PMA could increase the rate of NET formation. Evidence collected during the study showed that aLf enhanced NET formation. The changes were also linked to increased EGPA activity.
Yoshida et al. (2016) 27	Clinical study	The researchers reported that ANCAs were linked to small-vessel vasculitis due to its role in kidney damage and the formation of NETs. The study further showed that NET induction was correlated with increased ANCA affinity for MPO in patients with ANCA-associated microscopic polyangiitis. Therefore, the targeting of NET formation markers could help in managing microscopic polyangiitis.
Aleman et al. (2016) 28	Experimental study	The crosslinking of receptors like integrins did not enhance the NET formation process. It was also reported that the crosslinking of FcγRIIIb could induce NET formation in a way that is similar to the PMA-stimulation mechanism. Furthermore, the NET formation process was found to be dependent on ERK, PKC, and NADPH oxidase activation.
Aleyd et al. (2016) 29	Clinical study	The researchers stated that IgA immune complexes could activate neutrophils, a trend that shows their role in the joint damage and development of vasculitis. In addition, the blocking of FcαRI reduced the rate of neutrophil activation, thus highlighting the possible link between NETs and autoimmune disorders.
Sandin et al. (2016) 30	Clinical study	The study showed that the SIgA PR3-ANCA and IgA PR3-ANCA levels were higher during active ANCA-associated vasculitis than during inactive disease. The results of the study further suggest that SIgA PR3-ANCAs and IgA PR3-ANCAs are linked to disease activity in patients suffering from vasculitis. Additional research may be required to determine how the biomarkers and their isotopes can be used to monitor disease progression.
Kumar et al. (2015) 31	Clinical study	Kumar et al. (2015) noted that anti-histone IgG, heparin, and recombinant-activated protein were effective in annulling severe glomerulonephritis. Combination therapy did not have a significant additional effect on the severity of the disease. The results show that NET-linked histone release can be targeted to help in the treatment of glomerulonephritis.
Pieterse et al. (2017) 32	Experimental study	The results of the survey show that excessive NET formation can exceed the endothelial cell phagocytic capacity, thus causing vascular damage. In addition, it can lead to the activation of β-catenin signals and VE-cadherin degradation.
Westhorpe et al. (2017) 33	Animal model study	The *in vivo* imaging experimental results showed the transient presence of intraglomerular NETs, an indication that high shear conditions could disrupt the NETs in the glomerular capillaries. The researchers also reported that NET dissolution by DNase I did not change the glomerular injury. The findings show that NET generation is often enhanced during glomerulonephritis.
Söderberg and Segelmark (2018) 34	Review	NETs are involved in the development and progression of vasculitis. However, the NET formation process may also have a protective effect during the development of primary vasculitis.
van Dam et al. (2019) 35	Clinical study	The authors noted that the induction pathways and kinetics involved in NET formation differ in patients with AAV and SLE. The successful recognition of diversity in the NET formation process helps understand the pathological role of neutrophils in the progression and activities of different autoimmune disorders.
Lood and Hughes (2017) 36	Clinical study	The researchers stated that levamisole and cocaine are implicated in the development of ANCAs as they can induce the release of inflammatory NETS, BAFF, and NE autoantigens. The drug-associated release of CLAA-IgG is considered a reliable mechanism that shows the link between vasculitis and acute drug exposure among patients suffering from CLAA. Further investigations are needed to determine how the association can help in the management of ANCA-associated vasculitis and other disorders that are linked to NETosis.
Martinez et al. (2017) 37	Systematic review	The author stated that the successful identification of NET formation inhibitors is critical for the management of Net-dependent diseases. The phenotypic NET assay can be developed and used to diagnose diseases that are linked to NETosis, such as vasculitis.
Kolaczkowska et al. (2015) 38	Review	The study showed that neutrophils usually release NETs into the vasculature of the liver. DNase can effectively inhibit NE proteolytic activity. However, the molecule is incapable of removing the histones on the vessel walls, thus offering minimal protection against injury. The researchers further stated that the prevention or inhibition of NET formation could reduce the extent of tissue damage in the host during proteolytic activity.
Sondo et al. (2019) 39	Experimental study	The researchers conducted high-content imaging analysis and identified vanilloids as an effective chemical compound that can prevent NET release and NETosis induction. Furthermore, vanilloids could reportedly reduce ROSS production and stop excessive NET formation during autoimmune disorders.

**Table 2:** Summary of the findings

Event	Associated Disease
Dying neutrophils surrounding the walls of small vessels 45,46	Small-vessel vasculitis such as AAV 45,46
PR3-ANCAs or MPO-ANCAs are found in most patients with vasculitis 46	AAV pathogenesis 46
Presence of NET fragments in kidney biopsies 22,48	Henoch-Schonlein purpura, granulomatosis with polyangiitis, microscopic polyangiitis, and eosinophilic granulomatosis 22,48
Antibodies against PR3 24,57	Wegener's granulomatosis 24,57
Antibodies against MPO 24	Polyarteritis nodosa 24
Increased levels of MPO-ANCA IgG and PR3-ANCA IgG 58	AAV 58
Activation of neutrophils via Fcγ receptor IIIb 27,28	ANCA-associated microscopic polyangiitis 27,28
Presence of aggregated immune complexes 28	Vasculitis, SLE, and small-vessel leukocytoclastic vasculitis 28
High levels of PR3-ANCA IgA 30	SLE and rheumatoid arthritis 30
Vascular damage and endothelial cell apoptosis 30,66	AAV 30,66
Increased NET formation rate 33,68	Tubulointerstitial nephritis and glomerulonephritis 33,68
Activation of glomerular basement membrane (GBM) antibodies during NET formation 32,68	Tubulointerstitial injury and epithelial tubular cell hypoxia 32,68
Histone release during NET formation 30,66	Tubulointerstitial injury and AAV 30,66
